# Central Serous Chorioretinopathy in Endometriosis Treatment with Progestogen: A Metabolic Understanding

**DOI:** 10.3390/life15020144

**Published:** 2025-01-22

**Authors:** Francesco Chiara, Sarah Allegra, Maura Caudana, Jacopo Mula, Davide Turco, Simona Liuzzi, Maria Paola Puccinelli, Giulio Mengozzi, Silvia De Francia

**Affiliations:** 1 Laboratory of Clinical Pharmacology “Franco Ghezzo”, Department of Clinical and Biological Sciences, University of Turin, S. Luigi Gonzaga Hospital, 10043 Orbassano, TO, Italy; francesco.chiara@unito.it (F.C.); sarah.allegra@unito.it (S.A.); mauracaudana98@gmail.com (M.C.); 2 Laboratory of Clinical Pharmacology and Pharmacogenetics, Department of Medical Sciences, University of Turin, Amedeo di Savoia Hospital, 10149 Turin, TO, Italy; jacopo.mula@unito.it; 3 Health Local Authority “Asl City of Turin”—Ophthalmic Hospital, 10154 Turin, TO, Italy; davide.turco@aslcittaditorino.it; 4 Laboratory of Clinical Biochemistry “Baldi e Riberi”, Metabolic Diseases Unit, AOU Città della Salute e della Scienza di Torino, 10126 Turin, TO, Italy; simona.liuzzi@edu.unito.it (S.L.); mpuccinelli@cittadellasalute.to.it (M.P.P.); gmengozzi@cittadellasalute.to.it (G.M.)

**Keywords:** endometriosis, PR, chorioretinopathy, CSC, dienogest

## Abstract

Endometriosis afflicts 10% of women in their reproductive years and nearly half of women with infertility, and its etiology is not yet clear. Pharmacological therapy is generally based on progestins like progestogen. This drug binds to progesterone receptors with many known side effects. Here, we describe the case of a 33-year-old woman surgically treated for endometriosis who continued with drug therapy based on estradiol valerate and dienogest. Approximately 21 months after treatment, she reported ocular symptoms with vision alteration, diplopia, and metamorphopsia related to central serous chorioretinopathy (CSC). After the discontinuation of combined progestin-based treatment, the CSC fully subsided. Semeiological, clinical, and laboratory approaches were adopted, and urinary steroids were measured. A slight increase in prolactinemia in the absence of macro-prolactinemia was reported. The steroidal profile appeared without abnormalities, although a slight alteration of estrogen balance was noted. Considering the pharmacodynamics of dienogest versus selective progesterone receptor modulators, it can be assumed that patients’ clinical events are related to specific site response to steroids that bind the progesterone receptor. Dienogest may have induced the CSC as a not yet characterized side effect of the drug. Undoubtedly, further specific studies are needed concerning the metabolic and pharmacodynamic aspects that cannot be exhaustively covered here.

## 1. Introduction

Currently, endometriosis has a prevalence of approximately 10%, and from the knowledge in our possession is a multifactorial pathology, whose etiology is not fully clarified [1]. Endometriosis is characterized by endometrial glands and stroma in locations outside the endomyometrium. It occurs in as many as 10% of women in their reproductive years and in nearly half of women with infertility. It is a common cause of dysmenorrhea and pelvic pain and may present as a pelvic mass filled with degenerating blood (chocolate cyst) [2]. It is frequently multifocal and may involve tissue in the pelvis (ovaries, pouch of Douglas, uterine ligaments, tubes, and rectovaginal septum) and less frequently more remote sites of the peritoneal cavity and around the umbilicus and uncommonly lymph nodes, lungs, and even the heart, skeletal muscle, or bone [3,4]. Three possibilities (not mutually exclusive) have been invoked to explain the origin of these dispersed lesions. First, the regurgitation theory, currently the most accepted, proposes menstrual backflow through the fallopian tubes with subsequent implantation. Indeed, menstrual endometrium is viable and survives when injected into the anterior abdominal wall; however, this theory cannot explain lesions in the lymph nodes, skeletal muscle, or lungs. Second, the metaplastic theory proposes the endometrial differentiation of coelomic epithelium, which is the origin of the endometrium itself [5,6]. This theory too, cannot explain endometriotic lesions in the lungs or lymph nodes. Third, the vascular or lymphatic dissemination theory has been invoked to explain extrapelvic or intranodal implants. All pathways are conceivably valid in individual instances. The clinical manifestations of endometriosis depend on the distribution of the lesions. Extensive scarring of the oviducts and ovaries often produces discomfort in the lower abdominal quadrants and eventually causes sterility [7,8]. Pain on defecation reflects rectal wall involvement, and dyspareunia (painful intercourse) and dysuria reflect involvement of the urine and bladder serosa, respectively. In almost all cases, there is severe dysmenorrhea and pelvic pain as a result of intrapelvic bleeding and periuterine adhesions [9]. Current treatments for endometriosis encompass conservative surgery and fertility treatment, alongside hormone therapy, generally in combination with pain relievers [10]. Therapeutic choices are different, also based on the patient’s clinical situation, and include hormonal contraceptives that can help to control the hormones responsible for the buildup of endometrial tissue [11]; gonadotropin-releasing hormone (Gn-RH) agonists and antagonists, typically in combination with a low dose of estrogen that blocks the production of ovarian-stimulating hormones, limiting estrogen levels [12]; and progestin therapy that can stop the menstrual periods and the progression of endometrial formation and aromatase inhibitors with progestin to reduce the amount of estrogen [13,14]. Progestin-based therapy is chosen for the long-term treatment of endometriosis, and the first option is dienogest. It is an oral progestin that has been investigated systematically in Europe and Japan for the treatment of endometriosis in different clinical studies, from dose-ranging and placebo-controlled to active comparator-controlled, and long-term trials have been performed. Based on these studies, dienogest in monotherapy has received approval for the treatment of endometriosis in Europe, Australia, Singapore, and Japan [15]. Pharmacologically, dienogest grants the positive effects of both the 19-norprogestin and the progesterone derivative classes. When administered continuously, dienogest induces a hypoestrogenic, hypergestagenic local endocrine environment, leading to a decidualization of endometrial tissue with consequent atrophy of the endometriotic lesions. Animal studies suggest that dienogest may also lower plasma estradiol exposure via the apoptosis of granulose cells in the ovary [16]. Many studies in vitro and in animals show that dienogest has a direct inhibitory effect on the proliferation of endometrial-like tissue that is independent of progesterone receptor binding [17,18]. Another important mechanism of action for dienogest is the inhibition of angiogenesis, which is important in endometriotic lesions, and the molecular mechanisms forming the basis of this process continue to be explored [19,20]. Dienogest has been investigated as a long-term treatment of endometriosis in two large trials (Europe and Japan) evaluating efficacy, change in quality of life, safety, and tolerability. During the long-term study, laboratory parameters, vital signs, and body weight remained within range. Adverse treatment-related effects developed in 16.1% of women, including breast discomfort (4.2%), nausea (3.0%), and irritability (2.4%). The maximal intensity of treatment-related adverse events was mild or moderate in 92.5% of cases. The most reported treatment-related adverse event was metrorrhagia (71.9%), followed by headaches (18.5%) and constipation (10.4%). None of the treatment-related adverse events was rated as serious [21,22]. Currently, many mechanisms and possible negative implications of dienogest remains poorly explored. In this study, we presented the case of a 33-year-old woman with endometriosis treated with estradiol valerate/dienogest after surgical intervention. The patient developed central serous chorioretinopathy (CSC) during progestin treatment, with a complete regression after cessation of dienogest treatment.

## 2. Materials and Methods

### 2.1. Anamnesis and Diagnosis

This study presents a 33-year-old female patient diagnosed with a polyp of the uterus body and endometriosis cysts of the left ovary. The patient underwent resectoscopic and laparoscopic polypectomy surgery by the removal of endometriosis cysts. The surgery was conducted in the first instance with the positioning of anterior and posterior ibere valves with subsequent dilation by Hegar with an increasing number up to 10. Through the insertion of the resector, it was possible to visualize a regular cervical canal, regular isthmus, morphologically regular uterine cavity occupied by the endometrial polyp with left lateral implantation, regular and in situ osti, and hyperplastic endometrium. The intervention then continued with the removal of the endometrial polyp by means of a bipolar loop and slicing technique and with the removal of portions of endometrium. At this point, a uterine manipulator was positioned with the induction of pneumoperitoneum by means of a Verres needle, and subsequent introduction of three-way optics in the left iliac fossa, right iliac fossa, and suprapubic seat was carried out. In this context, the following were visualized: the uterus affected by the presence of voluminous nodes of fundic myoma; the right ovary, regular in shape and size; and the left appendage deformed by the presence of a massive cyst of about 8 cm.

The perforation of the cyst caused leakage of dense liquid with a “chocolate” appearance that is aspirated. The cyst was then removed by stripping of the cystic wall, and the tubal patency was subsequently verified by salpinge kromoscopy. In this phase, the tubal patency preserved on the left and the obstacle at the isthmic level on the right are highlighted. Endometriosis foci were also observed at the abdominal wall and perivesical peritoneum, on which diathermocoagulation was performed along with excision of the affected area. An intramural myoma node with a diameter of about 5 cm at the level of the uterine bottom was identified; regularity of the Douglas hollow, of the vesicular-uterine fossa, and of the annexes was observed. Myomectomy was then performed. An incision of the uterine wall was then performed at the myoma, highlighting the capsule of the myoma and its cleavage plane, before proceeding to its enucleation.

### 2.2. Pharmacological Treatment and Instrumental Exams

Histopathological examination of the intraoperative findings confirms the diagnosis of endometriosis on biopsy fragments of prevesical peritoneum. The subsequent pharmacological treatment of endometriosis involved the administration of estradiol valerate/dienogest at a low dosage over a 28-day cycle (see Table 1).

The treatment with estradiol valerate in combination with dienogest orally was administered with different dosages depending on the day of the month, completing a cycle of 28 days as shown in Table 1. This treatment continued for 21 months, with the appearance of ocular symptoms such as diplopia, blurred vision, and, in general, metamorphopsia.

**Figure 1:**
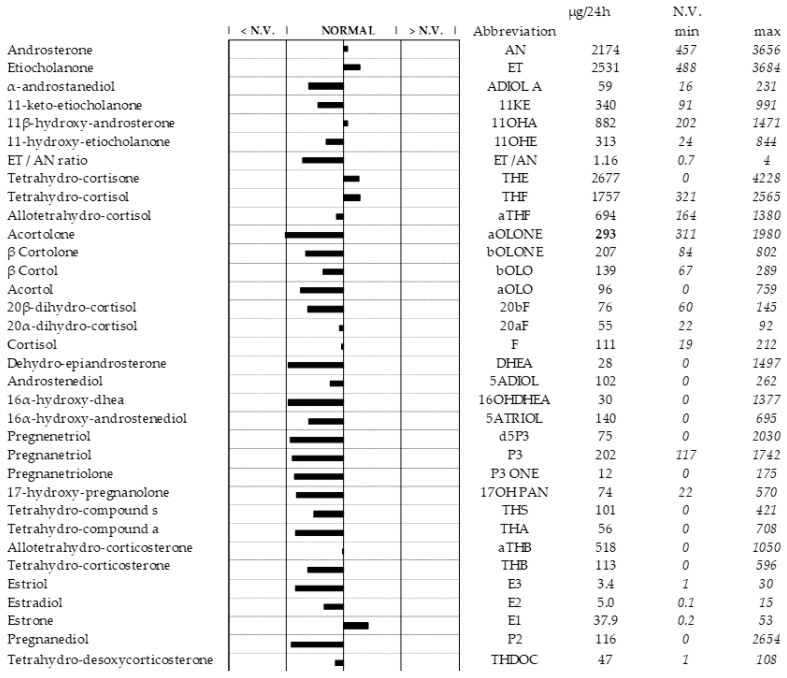
Patient’s steroid profile for sample of 24 h urine. Minimum and maximum ranges were obtained from subjects aged between 18 and 55 years. The amount of single metabolite was expressed in µg of compound normalized on the 24 h diuresis. [N.V. is the normal value].

Optical coherence tomography (OCT) was performed, and serous fluid from the choroid was infiltrated, with detachment localized between the inner/outer photoreception layer and the retinal pigmented epithelium (see Figure 1). This clinical picture led to the diagnosis of CSC.

### 2.3. Clinical Biochemistry

Clinical and hematological tests were carried out. Serum prolactin was dosed after the interruption of pharmacological therapy, and the steroidal profile was then determined from a 24 h urine sample using the gas chromatography method coupled to mass spectrometry (GC-MS), according to Shackleton et al. [23,24], to further deepen the metabolic condition.

To investigate the patient’s metabolic balance, the ratio of the steroids was investigated. Specifically, the ratio of concentrations of etiocholanolone [Et] and androsterone [An] and its reciprocal was considered and compared with reference values reported by Rousson V. et al. [25] to understand the equilibrium of the androgen pathway.

In the same way, the balance between testosterone biosynthesis pathways was evaluated, considering the ratio of pregnanediol (P2) to the sum of Et and An.

Furthermore, estrogen levels were evaluated through the ratio between estrone (E1) and the sum of 17β-estradiol (E2) and estriol (E3). The reference value in normal conditions for this ratio was not considered by Rousson in his work; thus, as part of our work, we analyzed the urine of 36 healthy women aged between 30 and 40 years with the GC-MS technique, reporting E1 and its relationship with the sum of the concentrations of E2 and E3. A comparison with values reported in previous studies revealed consistency for most ratios; however, minor deviations were noted, likely reflecting population and methodological differences. These findings highlight the need for standardized reference ranges across studies to enhance comparability and clinical applicability.

## 3. Results

The diagnostic evidence obtained from the anatomical–pathological examination of the intraoperative findings confirmed the diagnostic hypotheses based on the gynecological semeiotics of endometriosis. Surgical treatment allowed the removal of several endometriosis foci, including those at the bladder peritoneum.

Estradiol valerate/dienogest treatment provided clinical evidence of containment and subsequently abolition of the symptoms reported by the patient in the perioperative period, summarized as the total absence of heavy menstrual bleeding (HMB) and pelvic pain.

After 21 months from the beginning of drug treatment, the patient began to report ophthalmologic symptoms as regards the visual functionality of the right eye. The most evident signs were those related to diplopia and metamorphopsia.

**Figure 2:**
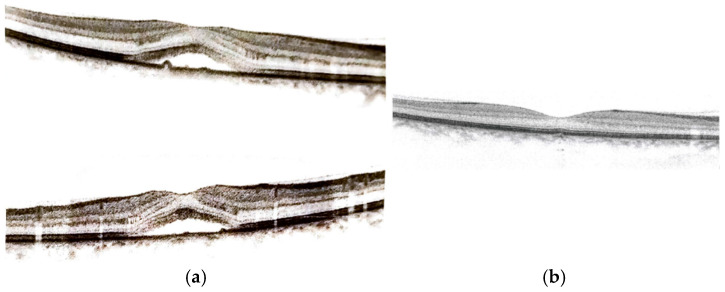
Optical coherence tomography (OCT) of the female patient: (**a**) infiltration of serous fluid from the choroid causing a small detachment of the inner/outer photoreception layer; (**b**) reabsorption of serous fluid and resolution of detachment after the interruption of treatment with estradiol valerate/dienogest.

Optical coherence tomography of the right eye showed a macular thickening due to a serus neuroretinal detachment. The photograph (in two sections) in Figure 2a reports an increased value of 371 µm of foveal thickness at the level of the central zone. This value returned to the norm (259 µm) 15 days after the discontinuation of drug therapy based on estradiol valerate/dienogest (see Figure 2b).

The morphology of the retinal layers and the increased BMO-MRW values led to the diagnosis of CSC, which progressively regressed with complete resolution of the visual symptomatology 15 days after the discontinuation of the aforementioned drug therapy. Possible correlation between progestin therapy and the onset of CSC was investigated. The hematochemical and hematological conditions, which did not provide any significant evidence, were therefore examined in depth. An increase in prolactinemia (41.2 µg/L) in the absence of macro-prolactinemia (estimated monomer/total ratio of 72%), however, was observed.

Apparently, the steroid profile was in the norm, although some variations are highlighted (Figure 2).

All ratio values to evaluate the functionality of some enzymes and their metabolic flow according to specific pathway are reported in Table 2 for a better understanding of the urinary steroid profile.

The [An]/[Et] ratio and its reciprocal were 0.859 and 1.164, respectively, and fell within the range of normal values.

Concerning testosterone biosynthesis balance, P2 to the sum of the Et and An ratio showed a slight decrease compared to the lower limit of normality (0.025 < 0.0376).

When considering estrogen balance, reference values for the ratio of E1 and the sum of the concentrations of E2 and E3 obtained from 36 healthy women are reported in Table 3.

The patient’s estrogen ratio was 4.51, above the calculated reference value.

## 4. Discussion

The patient’s steroid profile led to some potentially relevant considerations on the physio-pathological mechanisms that had a partial clinical response in the patient.

**Figure 3:**
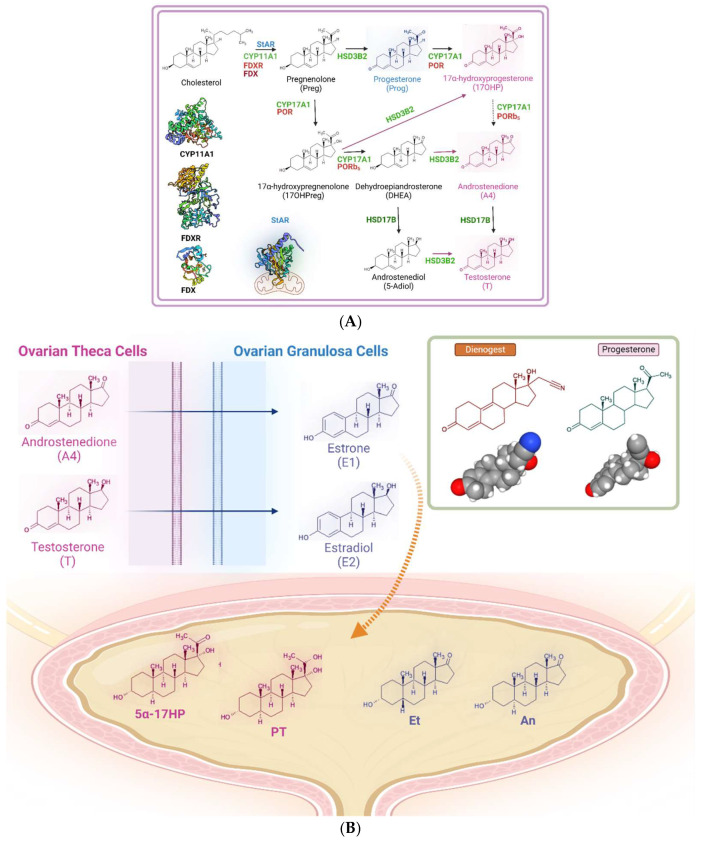
Ovarian theca cells and granulosa cell steroidogenesis (**A**). Urinary metabolites of androstenedione and testosterone; the top right pane shows the molecular structures of dienogest and progesterone, with space-filling representations (**B**). Abbreviations: cytochrome b5 (b5); cytochrome P450 oxidoreductase (POR); ferredoxin (FDX); ferredoxin reductase (FDXR); hexose-6-phosphate dehydrogenase (H6PDH); PAPS synthase 2 (PAPSS2); steroidogenic acute regulatory protein (StAR); 17OH-allopregnanolone (5α-17HP); pregnanetriol (PT); etiocholanone (Et); androsterone (An).

Considering the ovarian steroidogenesis (see Figure 3A,B), the two main pathways are the Δ4 pathway and the Δ5 pathway: the first reaction series leads to synthesis of progesterone, while the second metabolic process results in testosterone as the final metabolite in the ovarian theca cells (or in Leydig cells) [26]. The most prominent step in this method of steroid biosynthesis is the transport of cholesterol from the outer mitochondrial membrane to the inner membrane through the StAR (hormone-induced steroidogenic acute regulatory protein) [27]. The first rate-limiting step of steroidogenesis is the conversion of cholesterol into pregnenolone consisting of free, distinct biochemical reactions: 20α-hydroxylation, 22-hydroxylation, and the side-chain cleavage reaction (C–C bond cleavage of 20R,22R-dihydroxycholesterol) [28]. These facts suggest that the reactions involved in the synthesis of androstenedione (A4) and testosterone (T) are in a steady-state condition during physiological conditions [29]. In fact, the metabolic flux of these steroidogenic enzymes is basically unidirectional; therefore, the accumulation of a product does not promote the reverse reaction [30]. Consequently, when considering the basal (after the discontinuation of progestin-based therapy) steroid profile of the reported patient, it is essential to assess which pathway contributes more to the formation of Et and An to evaluate any “asymmetries” of such metabolism. The ratio [An]/[Et] gives information about the activity of 5alpha-reductase, while its reciprocal [Et/An] indicates whether this process is more oriented toward the androgen backdoor pathway (ABP) than the classical pathway [31,32]. These ratios, 1.16 and 0.859 (reported in Figure 2), respectively, fell within the range of normal values.

In general, these ratios, other than being pathological for certain diseases related to the defects of steroidogenesis, can be used as indices of evaluation of steroid metabolism.

Considering the pathways involved in testosterone biosynthesis, represented by Δ4 via progesterone or Δ5 through DHEA, the balance between these pathways could be evaluated considering the ratio of P2 to the sum of Et and An. In detail, the considered patient showed a slight decrease in this ratio compared to the lower limit of normality (0.025 < 0.0376). An opposite trend was observed when considering estrogen levels following evaluation of the ratio between E1 and the sum of E2 and E3.

In the works of Rousson V. et al. and Bochud M. et al., E1 and the relationship with its metabolites E2 and E3 are not considered; it is therefore difficult to compare E1 values or their relationships with other estrogens within reference values obtained by analyzing the urine of healthy women [33]. Shakleton et al. in their work reported E1 values, but few patients were considered, and distinction by age was lacking [34]. For this work, reference values for E1 to the sum of E2 and E3 were obtained analyzing the urine of 36 healthy women (Table 3).

The patient’s estrogen levels above the range limit indicated a shifted equilibrium toward the precursor of E2 and E3. This typically occurs at physiological progesterone levels that inhibit the increase in the concentrations of E2 and E3 that are the basis of pathological frameworks, like endometriosis [35]. Therefore, considering the fact that P2 levels at the time of the steroid dosing were physiological, it could be highlighted that the patient’s steroidogenesis occurred with a metabolic flow that exhibited predominant progestin activity with a shift in the estrogenic balance toward E1.

The diagnostic ratios of urinary steroid metabolites reported in Table 2 highlight several metabolic abnormalities that may provide insights into the underlying mechanisms linking estradiol valerate/dienogest therapy and central serous chorioretinopathy (CSC).

17,20-lyase Δ5-pathway deficiency:

The elevated ratios involving [d5P3] (e.g., [d5P3]/([DHEA] + [16OHDHEA]) = 3.480; range 0.028–1.880) indicate impaired activity of 17,20-lyase, a critical enzyme in the Δ5 pathway of steroidogenesis. This deficiency may result in a shift toward upstream Δ5 precursors and an overall increase in systemic estrogenic activity. Elevated estrogen levels could exacerbate choroidal vascular permeability and inflammatory processes, key contributors to CSC pathogenesis.

P450c17 global Δ4 vs. Δ5-pathway imbalance:

The reduced [P2]/([An] + [Et]) ratio (0.025; range 0.0376–1.140) suggests a preferential shift toward Δ4 steroidogenesis, enhancing progestin activity. Dienogest, as a potent progestin, may further modulate progesterone receptor (PR) activity in ocular tissues, disrupting vascular homeostasis in the retina and choroid.

5α-reductase activity:

While the [Et]/[An] ratio (1.164) falls within the normal range, slight deviations in [An]/[Et] (0.859; range 0.400–2.100) suggest minor shifts in androgen metabolism. This could influence androgen receptor (AR) signaling, which is critical for maintaining retinal homeostasis and choroidal stability. The dysregulation of AR activity has been implicated in vascular dysfunction, which may predispose to CSC.

These metabolic imbalances point to a complex interaction between progestogenic, estrogenic, and androgenic pathways in the reported patient. Enhanced systemic estrogenic activity, combined with increased progestin effects and altered androgen signaling, may destabilize the ocular microenvironment, leading to CSC. Future studies should investigate the direct impact of these metabolic abnormalities on steroid receptor modulation in ocular tissues, as well as their effects on choroidal vascular permeability and inflammatory responses.

All this considered, the retina and the choroid present progesterone (PR), androgen (AR), and estrogen (ER) receptors [36], demonstrating that the progesterone receptor is a ligand-activated transcription factor and member of the nuclear receptor (NR) superfamily. All nuclear receptors have three preserved functional domains, binding to DNA, the N-terminal domain, and the C-terminal domain, respectively, that bind to ligands [37]. When progesterone binds to its receptor, a conformational change occurs that transforms it into an active transcription factor. At this point, phosphorylation of the receptor occurs, but its role is not yet completely clarified.

The receptor then dimerizes to form PR-A and PR-B, which are not structurally identical. These isoforms have similar activities related to the steroid hormone–DNA interaction, but their functional activities are unique. Dimers interact with specific sequences of nuclear DNA (hormone-responsive elements) in genes that respond to progesterone. The dimer–agonist receptor complex is combined with coactivators that bind the complex to the transcription gene [38]. Thus, the rate of gene transcription is increased or decreased. Once gene transcription is started, proteins are synthesized, and then in the cells and organs, different physiological effects are observed.

These receptors, particularly PR and GR, play critical roles in the regulation of inflammation, vascular permeability, and oxidative stress, which are key factors in the pathogenesis of CSC.

The delayed onset of ocular symptoms in the reported case—manifesting only after 21 months of dienogest therapy—may be attributed to cumulative steroid exposure and the gradual dysregulation of choroidal and retinal homeostasis. Prolonged administration of dienogest could lead to the chronic modulation of receptor activity, eventually affecting choroidal circulation and retinal pigment epithelium (RPE) function. This process, combined with potential individual susceptibility factors such as genetic polymorphisms in receptors or metabolic genes, might explain the latency in symptom manifestation.

Supporting evidence from the literature indicates that chronic steroid exposure can lead to vascular dysregulation and increased choroidal permeability, both of which are central to CSC pathophysiology. Similar effects have been described in cases of corticosteroid-induced CSC, suggesting that even weaker glucocorticoid receptor activity, as seen with dienogest, could contribute to this adverse event over time [39,40].

These observations underscore the need for careful monitoring of patients on long-term dienogest therapy for potential ocular side effects, especially in individuals with predisposing factors such as choroidal vascular instability or a history of CSC.

While this study primarily focused on the progestin-related effects of dienogest, estradiol valerate, a synthetic estrogen, may also play a contributory role in the development of central serous chorioretinopathy (CSC). Estradiol valerate undergoes rapid conversion to estradiol, which has been shown to influence intraocular pressure (IOP) and choroidal vascular permeability. According to Vajaranant et al. [41], estrogen therapy can significantly elevate IOP, a recognized risk factor for CSC. Elevated IOP could disrupt choroidal hemodynamics, leading to increased vascular permeability and serous retinal detachment.

Additionally, estrogen’s pro-inflammatory and pro-angiogenic effects might potentiate the steroidogenic environment created by dienogest, amplifying the cumulative impact on retinal and choroidal tissues. Notably, estrogen receptors (ERα and ERβ) are present in the retina and choroid, where they regulate vascular and metabolic homeostasis. Excessive activation of these receptors by estradiol could destabilize the blood–retina barrier, further predisposing the patient to CSC.

The interplay between estradiol valerate and dienogest may thus create a synergistic environment that exacerbates choroidal vascular dysfunction. Future studies should investigate the individual and combined effects of these agents on ocular physiology to better understand their role in CSC pathogenesis.

The main physiological site of action of progesterone is the uterus, although its receptor, like that of other steroid compounds, is found in different anatomical districts, including retinal tissue and the choroid. It should therefore be highlighted that progestins, like dienogest, related to norethindrone and classified as analogues of testosterone, have a decidedly important role in producing events with unusual pharmacodynamics. Dienogest is structurally unique, presenting the molecular structure of estrane, a cyanomethyl group in C17 that replaces the ethylene group, and an unsaturated Δ9 (Figure 3). It is often used in combination with estradiol valerate to intervene in menopause disorders and has long since been utilized in the treatment of endometriosis, as in this case.

Focusing on the pathological manifestation linked to chorioretinopathy, one last piece of evidence should be considered. Nielsen J. S. et al. described in 2007 the case of a 63-year-old male patient with CSC, treated to full regression with mifepristone at a dose of 200 mg/day [42,43]. This leads to an interesting question: can a PR modulator like mifepristone treat CSC considering that a progestin like dienogest may have induced it in the reported patient? Based on the observation of the presented work, both mifepristone and dienogest bind to the PR and glucocorticoid receptor (GR), both belonging to the NR superfamily. Studies in which the affinity of dienogest for different NRs, like PRs, ARs, ERs, GRs, and mineralocorticoid receptors (MRs), has been experimentally measured are lacking in the literature; nevertheless, Robin-Jaerschmidt et al. [44], by performing site-specific substitutions in the amino acid sequence of GR and PR through a chimerical receptor called GPn, showed that it is possible to modify the affinity of several ligands, including mifepristone, with different receptors, thus obtaining equally different response activities.

For instance, in the GR, substitutions at residues Leu752, Glu755, and Met560 within the LBD have been shown to alter the receptor’s binding affinity for synthetic ligands, including mifepristone. Leu752→Ala substitutions, in particular, reduce ligand-binding affinity, while Glu755→Gln enhances selective ligand interactions.

In the PR, residues such as Asn719, Gln725, and Thr894 within the hinge region and the LBD are crucial for the receptor’s interaction with dienogest. Substitutions at Asn719→His or Gln725→Leu significantly affect the receptor’s conformational dynamics, altering its transcriptional activity.

These sequence-specific substitutions demonstrate the structural flexibility of nuclear receptors and their ability to accommodate ligand-specific conformational changes. Dienogest, due to its unique structural modifications (e.g., Δ9-unsaturation, cyanomethyl group at C17), may exploit these receptor dynamics differently from other progestins, thereby contributing to its distinct pharmacological profile.

Furthermore, the differential affinity of dienogest and mifepristone for other nuclear receptors (e.g., ERs, ARs, and MRs) requires further study. Quantitative evaluations such as relative binding affinity (RBA) and inhibition constant (Ki) across nuclear and membrane-bound receptor subtypes would provide critical insights into their pharmacodynamic properties. In particular, the role of membrane-bound progesterone receptors (mPRs) in mediating non-genomic rapid effects remains underexplored and warrants investigation [45].

A different modulation by mifepristone and dienogest on both the PR and the GR is therefore plausible. The predominant action of the antiglucocorticoid type exercised by mifepristone [46] resulted in a reduction in the inflammatory process with CSC resolution, while weak activity of dienogest on the GR, linked to a predominant progestin effect within the already highlighted steroid profile, led to an inflammatory effect that, at that moment, was only clinically describable with the appearance of CSC and complete resolution following therapy interruption.

The possible role of genetic polymorphisms in genes involved in estrogen activity could at least influence dienogest activity and related toxicities. Different polymorphisms have been reported in *ER1* and *ER2* genes [47,48]. Follicle-stimulating hormone (FSH) levels rise in the early part of the cycle, which raises estrogen levels. FSH, progesterone, and androgen levels all fluctuate during the menstrual cycle in addition to estrogen levels. Consequently, it is necessary to look into the genes for the AR, progesterone receptor (PGR), and FSH receptor (FSHR) as possible sources of genetic susceptibility. The two protein isoforms of the PGR gene, isoform A (PRA), preventing the activation of the estrogen receptors, and isoform B (PRB), activating the estrogen receptors, can alter the biological effect of progesterone and thus maybe also dienogest activity [49]. Like the ESR, the PGR participates in a number of intracellular signaling pathways and can be activated without a ligand. The ligand binding and the complete signaling cascade appear to be affected by a particular variant in the PGR gene known as PROGINS [50]. Additionally, of interest is the sex hormone-binding globulin (SHBG) gene, which produces a glycoprotein that binds to both estrogen and androgens. Cytochrome P450 A1 (CYP19A1), which converts testosterone to estrogen, is necessary for the last stage of estrogen production, and the CYP17A1 enzymes are essential for androgen production [51]. Lastly, CYP1A1 accelerates the hydroxylation of estrogen in extrahepatic tissues [52]. Catechol–estrogen or its products, such as nuclear receptor interacting protein 1 (NRIP1) and catechol-O-methyl-transferase (COMT), may also be influenced by other genes involved in estrogen metabolism. The transcription of estrogen receptors, especially ESR1, is adversely regulated by NRIP1, and COMT catalyzes O-methylation and hence deactivates estradiol metabolites [53,54].

## 5. Conclusions

The affinity of mifepristone and dienogest for different types of receptors should be further and deeper explored. Different pharmacodynamics and pharmacokinetics aspects need to be clarified, focusing on the understanding of the biochemical mechanisms underlying the clinical evidence described in this work. All the clinical and biochemical information reported in this work can uniquely justify the pathological manifestations observed though does not lead to a decisive conclusion regarding all mifepristone implication. The next step of our work will be the evaluation of dienogest metabolites to determine whether a specific alteration in dienogest metabolism is associated with CSC development.

## Figures and Tables

**Table 1:** Composition of estradiol valerate/dienogest tablets depending on the days of the month.

Day Number	Estradiol Valerate [mg]	Dienogest [mg]
1–2	3.0	0.0
3–7	2.0	2.0
8–17	2.0	3.0
18–24	2.0	3.0
25–26	1.0	0.0
27–28	0.0	0.0

**Table 2:** Diagnostic ratios of urinary steroid metabolites.

Enzymes and Pathways	Ratios	Value	Range
17β-hydroxysteroid dehydrogenase (HSD17B)	$\frac{\left[Et\right]+\left[An\right]}{\left[THE\right]+\left[THF\right]+\left[aTHF\right]}$	0.917	0.366–2.580
Alternative androgen backdoor pathway vs. classic pathway	$\frac{\left[An\right]}{\left[Et\right]}$	0.859	0.400–2.100
5α-reductase deficiency	$\frac{\left[Et\right]}{\left[An\right]}$	1.164	0.470–2.400
17,20-lyase Δ^5^-pathway deficiency	$\frac{\left[d5P3\right]}{\left[DHEA\right]+\left[16OHDHEA\right]}$	3.480	0.028–1.880
	$\frac{\left[d5P3\right]}{\left[5ADIOL\right]}$	1.980	0.166–5.510
	$\frac{\left[d5P3\right]}{\left[5ATRIOL\right]}$	1.440	0.0229–1.870
	$\frac{\left[d5P3\right]}{\left[DHEA\right]+\left[16OHDHEA\right]+\left[5ADIOL\right]+\left[5ATRIOL\right]}$	0.670	0.0097–0.555
P450c17 global Δ^4^ vs. Δ^5^-pathway	$\frac{\left[11OHA\right]}{\left[DHEA\right]+\left[16OHDHEA\right]}$	15.21	0.147–6.20
	$\frac{\left[11OHA\right]}{\left[5ADIOL\right]}$	8.65	1.08–20.4
	$\frac{\left[P2\right]}{\left[An\right]+\left[Et\right]}$	0.025	0.0376–1.140

Abbreviations: ET, etiocholanone; AN, androsterone; DHEA, dehydroepiandrosterone; 16OHDHEA, 16α-hydroxy-dehydroepiandrosterone; d5P3, pregnenetriol; 5ADIOL, androstenediol; 5ATRIOL, androstenetriol; 11OHA, 11β-hydroxy-androsterone; P2, pregnanediol.

**Table 3:** E1 and [E1]/{[E2] + [E3]} ratios. N represents the number of subjects included in the study as the control, and the third column reports the reference interval obtained by 2.5th–97.5th percentiles.

Analyte or Ratio	N	Reference Interval
E1	36	2.00–15.00
$\frac{\left[E1\right]}{\left[E2\right]+\left[E3\right]}$	36	0.20–3.50

## Data Availability

The original contributions presented in this study are included in the article. Further inquiries can be directed to the corresponding author.
